# The use of a portable ultrasound system in the surgical assessment of rib fractures in an elderly patient

**DOI:** 10.1016/j.amsu.2018.10.017

**Published:** 2018-10-16

**Authors:** Vidmi Taolam Martin, LiYuan Zeng, Jean Christian Nzengue, LiYe Mao, JingYin Huang, XiuFan Peng

**Affiliations:** a Department of Cardiovascular Surgery, Clifford Hospital, 3 Hongfu Road, Panyu, Guangzhou, Guangdong Province, PR China; b Department of Orthopedic Surgery, Clifford Hospital, 3 Hongfu Road, Panyu, Guangzhou, Guangdong Province, PR China

**Keywords:** Case report, Elderly, Rib fractures, Portable ultrasound system

## Abstract

**Introduction:**

Portable ultrasound is a modality of medical ultrasonography that utilizes small and light devices, and is an established diagnostic method used in clinical settings such as Cardiology, Vascular Surgery, Radiology, Endocrinology, Pediatric and Obstetric & Gynecology.

**Presentation of cases:**

We present a case report of 86-years old patient who underwent surgical rib fixation for multiple rib fractures followed by falling from standing height and our management experience.

**Discussion:**

The use of portable ultrasound device in operation theatre demonstrates several advantages.We believe that Portable color doppler ultrasound system would be necessary in the management of rib fracture.

**Conclusion:**

This study demonstrates that the portable ultrasound system is a valuable method of imaging in the assessment of rib fractures, and which can save time, economically affordable for many patients, and allow surgeons to make a minor incision in order to avoid complications such as infection, particularly in this group of vulnerable patients.

## Highlights

• Portable ultrasound is a modality of medical ultrasonography.
• Portable color doppler ultrasound system guiding might be valuable in assess-ment of rib fracture.
• Elderly patients are highly susceptible to rib fractures after trauma.

## 1 Introduction

A rib fracture is recognized to be one of the most frequent complications of chest traumas. It has been reported in the literature that a fractured rib can injure neighboring organs including the descending aorta, which is a life-threatening complication [1,2]. Data from the literature supports that approximately 10% of all blunt force injuries that occur in the elderly have had more than 1 rib fracture. The elderly patient, older than 65 years, was at high risk for rib fractures due to falls sustained from a standing position or from height such as climbing a ladder [3]. Several studies in the adult population of patients revealed that apart from associated injuries, other independent factors that adversely affect mortality rate include older age and higher number of fractured ribs [4,5]. Here, we reported the utility of a Portable color doppler ultrasound system mindray z5 in the management of rib fractures.

The first portable ultrasound machines arrived in the early 1980s but battery powered systems that could be easily carried did not arrive until the late 1990s. In November 2015 [6], Philips launched a new era of portable ultrasound when it came out with the Lumify portable ultrasound machine. In 2016, Clarius Mobile Health launched a wireless portable ultrasound scanner that is compatible with iOS and Android devices. The Clarius ultrasound scanner has Point-and-Shoot Ultrasound™ [7] capabilities, which optimizes common settings used by medical professionals at the point of patient care. But in our knowledge few cases of rib fracture under ultrasound system guidance in an operation theatre have been reported. In order to avoid the possible risk of atelectasis and pneumonia, breathing problems and pneumothorax or hemothorax, specially in those vulnerable group of patients, we decided to use a Portable Ultrasound system with a high frequency linear probe accessible easily to bone in the management of rib fractures to reach an acceptable level of surgery with a minimal surgical incision site. Our institutional ethics committee determined that approval was not necessary for a case report and the work has been reported in line with the SCARE criteria [8].

## 2 Clinical case

**Fig. 1:**
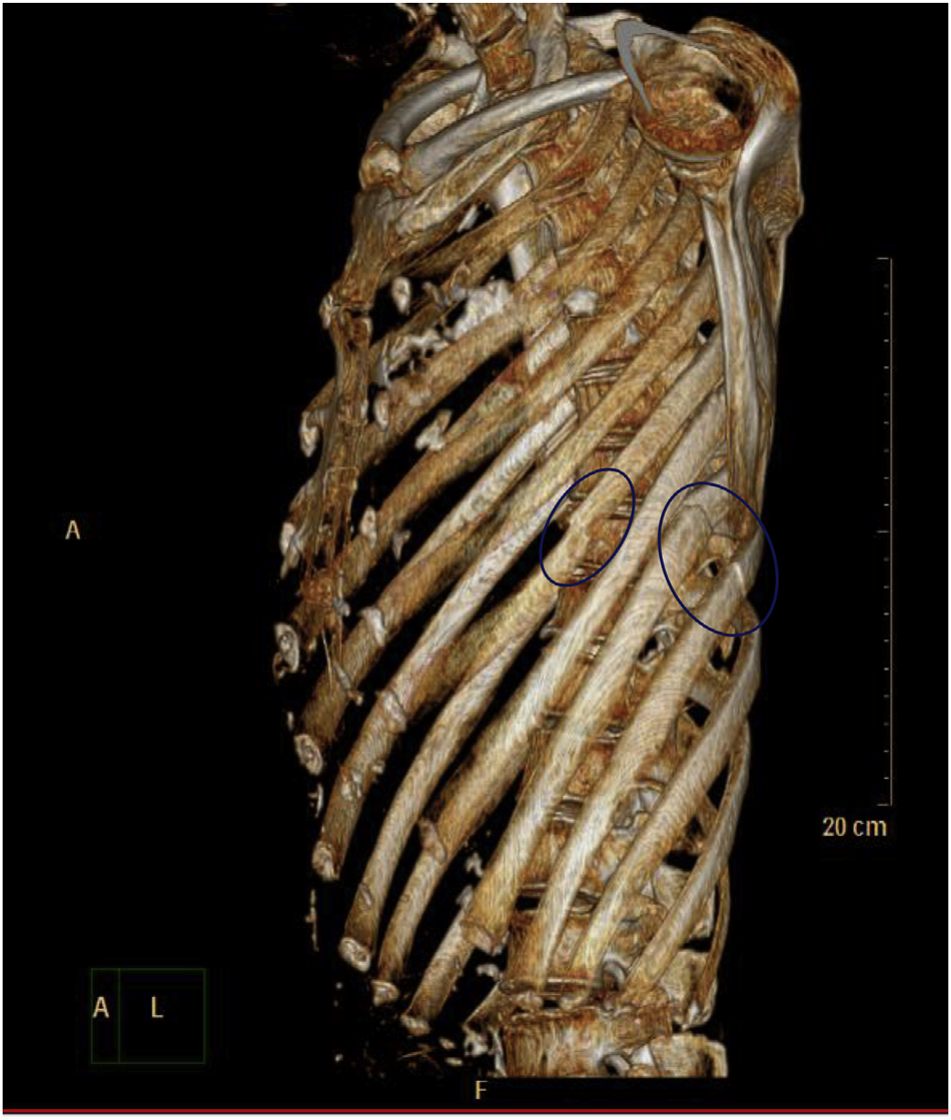
Thoracic imaging with rib fractures.

**Fig. 2:**
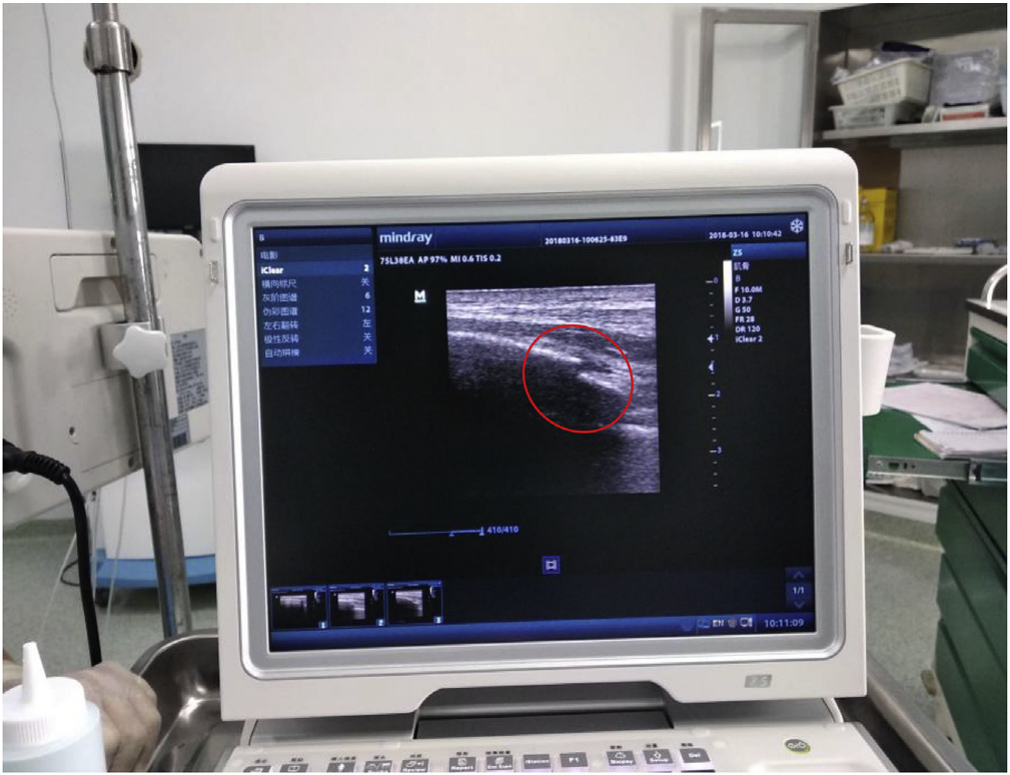
Ultrasound Image localizing rib fracture.

**Fig. 3:**
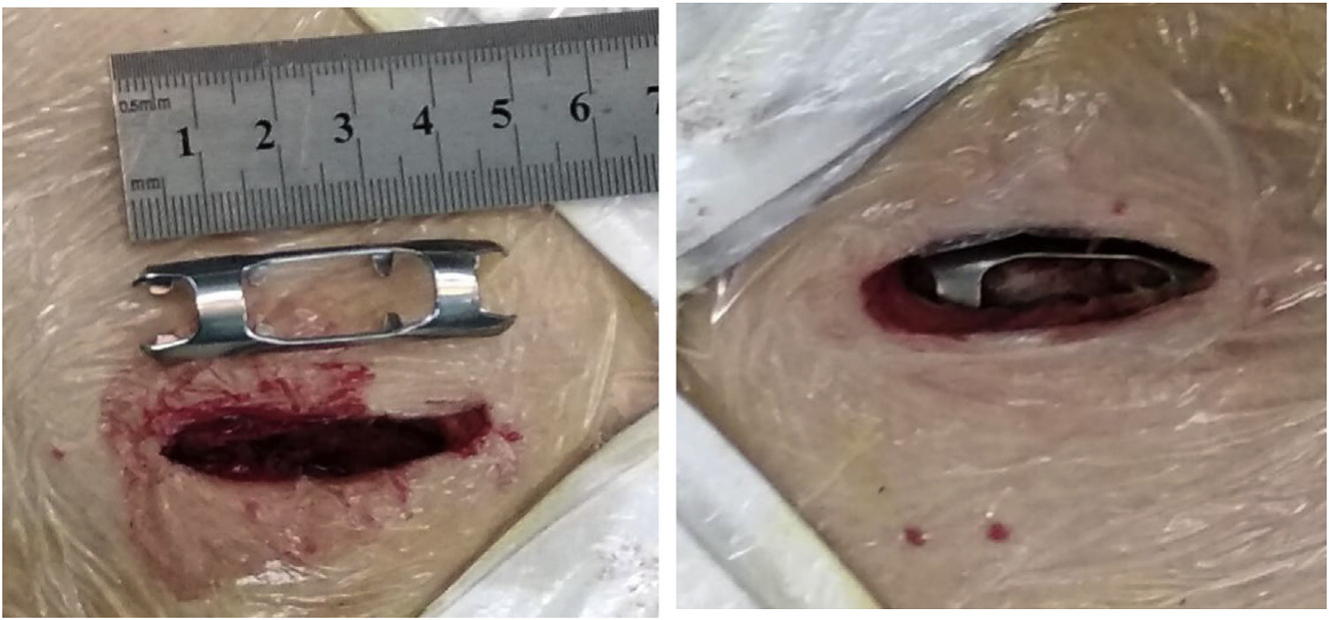
Surgical site incision(4cm) and internal fixator device placement.

A 86-year-old man presented to our Emergency Department after falling from standing height. Patient was immediately admitted from the Emergency Department to our trauma ward. On observation, the patient was noted to have a patent airway, decreased breath sounds and tenderness on the left chest, dyspnea with chest pain and the blood oxygen saturation level was decreased to 93% with room air, whereas hemodynamic measurements were stable. The patient whole body examinations did not reveal other injuries outside of the chest area. The Chest imaging revealed multiple rib fractures. In addition, computed tomographic scan examination showed comminuted fractures of ribs 6 through 9 on the left side, without lung contusion (Fig. 1), which we considered automatically to indicate operation in order to avoid the risk of abdominal organs injuries. Twenty four hours (24h) after injury, the patient underwent internal fixation of left ribs 8 and 9, An operation was then performed using a Portable color doppler ultrasound system mindray z5(Fig. 2) to localize the fractured rib. The patient was under general anesthesia with differentiated ventilation, and then he was placed into a right lateral decubitus position. Judet struts were used in the fixation of ribs fractures in our present study (Fig. 3). After 1hour, the operation ended successfully and the patient were moved to the ward. The patient was given a combination of oral and transdermal pain medications. At 5days after surgery, the patient's chest tube was removed. The reporting pain intensity were 8 of 10 for both rest and activity. Fifteen days after surgery, the patient was discharged from the hospital without complications. At 6weeks follow-up, the patient did not present any signs of chest pain or difficulty breathing on exertion.

## 3 Discussion

In comparison to the younger patients, it has been known that the elderly patient represent a higher rate of morbidity and mortality after sustaining rib fractures. In this present case report, we demonstrated that portable ultrasound system guided might be suitable for the management of rib fractures in those vulnerable group of elderly patients. The portable Ultrasound device can definitely save time and minimize the incision site from patients known to present unstable health conditions. Furthermore, portable ultrasound guidance allows the surgeon to visualize with precision the bone structures. The location of the rib fracture is extremely important because this leads to identifying injuries under the ribs. As we know, an ultrasound is being used increasingly in clinical diagnosis throughout the world in many medical specialties. In our case, a high resolution ultrasound system Mindray z5 was established. The device was able to provide anatomic information. According to data from literature, the choice of the ideal approach for rib fracture is critical to undertaking safe, effective operations. In addition, an incorrect incision choice with resultant inadequate exposure may lead to an unnecessary or difficult, ineffective operation or even fatal intraoperative complications. So, it has been stated that surgeons need to be familiar with the advantages and disadvantages of many different incisions. Fortunately, with the availability of CT scan, or Portable color Doppler Ultrasound system Mindray z5 potential intraoperative anatomic issues can be observed in advance, so that the planning phase can include alternative incisions or extensions to the initial approach if necessary.

Anatomically speaking, the thoracic cavity envelops the heart and lungs which are protected by the clavicles, sternum and ribs. But when a rib is fractured, it can perforated easily the lung organ and cause a major complication like pneumothorax. A similar study revealed that elderly patients sustaining rib fractures have unique features group, mainly secondary to lower bone densities, leading to higher bone fragility. They resume that despite lower injury impact required to cause rib fractures, these patients are in high risk of lung complications and have a higher mortality rate, probably secondary to lower lung compliance and high incidence of background comorbidities [[7], [8], [9], [10]]. It has been reported in another findings that mortality of rib fracture trauma victims is mostly affected by the presence of extrathoracic injuries and not associated intrathoracic injuries [11]. Such was not found in this current case report. But we believe that the exact localization of the fractured rib by the portable ultrasound device guided in the operation theatre would be very important in order to identify not only the fractured rib but also all probable injured internal organs.

The use of portable ultrasound device in operation theatre demonstrates several advantages. Technically, the device is able to visualize the bone and certain organs, such as lung, heart and vascular structures. For that reason, it would be valuable to use a portable ultrasound device to assess the majority of patients. On the other hand, minimally invasive procedure of the rib fracture can be assessed on high-risk patients under portable ultrasound system guiding. Therefore, portable ultrasound system is an ideal alternative, which may provides full anatomic information for treating high-risk patients. Performing a smaller incision can reduce bleeding, scarring and specially the risk of infection. Additionally, the advantage of the ultrasound device was to get less trauma, better cosmetic results and better postoperative course. Further studies are encouraged to evaluate the efficacy of ultrasound system in managing rib fractures.

In our knowledge, there are no studies that perform the rib fracture surgery under a portable color doppler ultrasound system guiding in the operation theatre, especially in an elderly patient with more than 80 years old. The present case demonstrates also the need for ultrasound device in term of avoidance of major incision, which can minimize post-surgery infection.

## 4 Conclusion

We concluded that a Portable color doppler ultrasound system might be an ideal tool in the management of rib fracture; It represent a time-saving in order to allow a substantial saving in terms of man-hours, economic and applicable method of imaging.

## Provenance and peer review

Not commissioned externally peer reviewed.

## Ethical approval

Our institutional ethics committee determined that approval was not necessary for a case report.

## Sources of funding

No source of funding or sponsors.

## Author contribution

**Vidmi Taolam Martin**: Participated substantially in conception, design, and execution of the study and in the analysis of data; also participated substantially in the drafting of the manuscript.

**LiYuan Zeng**: Participated substantially in conception, design, and execution of the study and in the analysis and interpretation of data.

**Jean Christian Nzengue**: Participated substantially in execution of the study and in the analysis and interpretation of data.

**LiYe Mao**: Participated substantially in execution of the study and in the analysis and interpretation of data.

**JingYin Huang**: Participated substantially in the drafting and editing of the manuscript.

**XiuFan Peng**: Participated substantially in conception of the study; also participated substantially in the editing of the manuscript.

## Conflicts of interest

All authors disclose no financial and personal relationships with other people or organisations that could inappropriately influence (bias) their work.

## Research registration number

None (non-human study).

## Guarantor

XiuFan Peng.

Vidmi Taolam Martin.
